# COVID-19-Associated Mucormycosis: Outcomes From a Tertiary Care COVID-19 Hospital

**DOI:** 10.7759/cureus.87104

**Published:** 2025-07-01

**Authors:** Vijaya Kumar Lukka, KVS Kumar Chowdary, Sri Lekha Bandaru, Kolli Naga Saritha

**Affiliations:** 1 Otolaryngology, NRI Institute of Medical Sciences, Vijayawada, IND; 2 Community Medicine, NRI Institute of Medical Sciences, Vijayawada, IND

**Keywords:** covid-19, covid-19-associated mucormycosis, long-term outcomes, mucormycosis, outcomes, risk factors

## Abstract

Background

Mucormycosis is a type of invasive fungal infection seen in diabetics and immunocompromised individuals. During the COVID-19 pandemic, there was a rapid surge in cases of mucormycosis, leading to the origin of the term COVID-19-associated mucormycosis (CAM). Although several studies described its outcomes after treatment, very few described both short-term and long-term outcomes. This study aims to describe the immediate and long-term outcomes of the treatment of CAM at NRI General Hospital (NRIGH), a tertiary care COVID-19 hospital, to identify the factors that affect the outcomes of treatment.

Methodology

This is a retrospective chart review of CAM patients treated at the Department of ENT, NRIGH, from November 2020 to January 2022. Data regarding demographics, comorbidities, symptomatology, extent of disease, extent of surgery, antifungal therapy, and outcomes of treatment after one, three, six, and 12 months were collected. Cure and stable disease were defined as successful outcomes. Unstable disease or mortality due to disease was defined as unsuccessful outcomes. Univariate and multivariate longitudinal ordinal logistic regression analysis was performed to assess the association between time, clinical characteristics, and treatment-related factors with an ordinal outcome measured longitudinally (baseline, three, six, and 12 months).

Results

There was no statistically significant association between age, sex, duration of symptoms, laterality of disease, steroid use, and CAM outcomes. There was a statistically significant association between diabetes, hypertension, baseline extent of disease, particularly intracranial extension, and outcomes of CAM treatment (p < 0.05). Extent of surgery exhibited a time-dependent trend, with strong associations only in univariate analysis at one month (p = 0.001) and three months (p = 0.005), but weakening associations at six and 12 months (p = 0.33). Longitudinal regression model showed that antifungal therapy with amphotericin B (AMB) was related to much poorer outcomes (odds ratio = 23.2, p = 0.001). Posaconazole (PCZ) alone or AMB followed by PCZ therapy showed trends toward better outcomes, with the latter regimen showing borderline statistical significance (p = 0.05), well-supported by univariate analyses at three, six, and 12 months (all p = 0.001). The all-cause mortality rate was 14.28%. Short-term (three months) and long-term (one year) treatment success was 85.71%.

Conclusions

Diabetes and the extent of baseline disease adversely affect the outcomes of treatment. Surgical technique likely plays a determining role in early recovery patterns, but becomes less determinant of long-term outcomes. Drug regimens and comorbidity burden may affect outcomes, but require larger studies for confirmation.

## Introduction

Mucormycosis is a type of invasive fungal infection usually seen in patients with diabetes, as well as those undergoing chemotherapy, immunosuppressive therapy, and dialysis [1]. It is caused by the fungi belonging to the subphylum Mucormycotina [2]. With the onset of the COVID-19 pandemic caused by SARS-CoV-2, there was a sudden surge in cases of rhino-orbito-cerebral mucormycosis, leading to the origin of the term COVID-19-associated mucormycosis (CAM), which is defined as patients with mucormycosis along with acute or recent (three months) COVID-19 illness [3]. Although several studies have been conducted on CAM, very few have described its immediate and long-term treatment outcomes. Hence, this study aims to describe the immediate and long-term outcomes of the treatment of CAM at NRI General Hospital (NRIGH), a tertiary care COVID-19 hospital, to identify the factors that affect the outcomes of treatment.

## Materials and methods

This is a retrospective chart study of all patients with CAM who were treated at NRIGH, a tertiary care COVID-19 hospital, from November 2020 to January 2022. Charts of patients whose data were incomplete were excluded from the study. Data on characteristics such as age, sex, symptoms of CAM, mean symptom duration, and use of systemic steroids during COVID-19 were collected. Data on patient comorbidities such as diabetes (new onset or pre-existing), hypertension, bronchial asthma, renal failure undergoing dialysis, and whether on chemotherapy or immunosuppressive therapy were collected. Data regarding the extent of disease, the extent of surgery, and the duration and total dose of antifungal therapy were also collected. Duration of follow-up and immediate (one month, three months) and long-term (six months, 12 months) treatment outcomes of CAM were also collected. Cure was defined as the complete resolution of disease (clinically, endoscopically, and radiologically, wherever feasible). Stable disease was defined as the complete resolution of symptoms clinically with significant regression of disease or non-progressive disease on nasal endoscopy or imaging. Unstable disease was defined as significant residual disease with worsening symptoms and progressive disease on nasal endoscopy or imaging [4]. Cure and stable disease were considered successful outcomes [5]. Unstable disease or mortality was considered an unsuccessful outcome. The data were analyzed by statistical methods to identify the factors that affect the outcomes of treatment.

Statistical analysis

All statistical analyses were conducted using R Studio (version 2024.02.0; Vienna, Austria). Categorical variables were expressed in terms of frequencies and percentages, and their associations with outcomes were analyzed using the chi-square test of association. A cumulative link mixed-effects model was used to determine the association between time, clinical characteristics, and treatment-related factors with an ordinal outcome measured longitudinally (baseline, three, six, and 12 months). Data were imported into R from a structured Excel dataset containing patient-level longitudinal information. The model included a random intercept for patient ID to account for repeated measures (n = 49 patients; 196 total observations). The cumulative link mixed model was fitted using the ordinal and lme4 packages, leveraging the Laplace approximation for maximum likelihood estimation. Diagnostic checks and inference were further supplemented using base and tidyverse tools.

## Results

A total of 61 CAM patients were treated during the study period. After excluding 12 records with missing or incomplete data, records of 49 patients with CAM were included in the study, and their data were analyzed. The age of the patients ranged from 30 to 80 years, with a mean of 52.08 years. Overall, 81% of patients were males and 18.36% were females. The duration of symptoms ranged from 2 to 90 days, with a mean of 16.37 days. The most common symptom seen was facial pain or swelling, followed by headache (Table 1). Many patients had multiple symptoms.

**Table 1:** Symptoms of patients.

Symptoms	n (%)
Nasal obstruction with discharge	16 (32.65%)
Facial pain or swelling	39 (79.59%)
Palate ulcer	6 (12.24%)
Gingival swelling and loosening of teeth	17 (34.69%)
Orbital involvement	18 (36.73%)
Loss of vision	8 (16.32%)
Headache	30 (61.22%)

Of 49 patients, 10 (20.40%) did not use steroids, and 39 (79.60%) used steroids during COVID-19 infection. Of the 39 who used steroids, 30 used them for more than five days, and only nine used them for fewer than or equal to five days. Of the 49 patients, 32 (65.30%) had pre-existing diabetes, whereas 13 (26.53%) were newly diagnosed diabetics during COVID-19 infection, and four were non-diabetics (Table 2). Overall, 22 (44.9%) patients had hypertension, five (10.2%) had coronary artery disease, and nine (18.36%) had other comorbidities such as hypothyroidism, bronchial asthma, Parkinsonism, and cerebrovascular accident.

**Table 2:** Risk factors.

Predisposing factor (N = 49)	n (%)
Steroid use during COVID-19	39 (79.60%)
Diabetes/Hyperglycemic state	45 (91.83%)
Pre-existing diabetes	32 (65.30%)
Newly diagnosed diabetes	13 (26.53%)
Hypertension	22 (44.90%)
Coronary artery disease	5 (10.20%)
others	7 (14.30%)

Further, 25 (51.02%) patients had bilateral disease, and 24 (48.98%) had unilateral disease at the time of presentation. The disease involved the nose alone (stage I) in only one (2.06%) patient, the nose and paranasal sinuses (stage II) in 23 (46.9%) patients, extended to the orbit (stage III) in 18 (36.73%) patients, and extended intracranially (stage IV) in seven (14.28%) patients (Table 3). All seven patients with intracranial extension also had simultaneous orbital disease.

**Table 3:** Extent of disease.

Extent of disease (n = 49)	n (%)
Nose alone (stage I)	1 (2.06%)
Nose and paranasal sinuses (stage II)	23 (46.93%)
Nose, paranasal sinuses, and orbit (stage III)	18 (36.73%)
Intracranial (stage IV)	7 (14.28%)

Further, 25 (51%) patients had orbital extension of the disease. In total, 17 (34.7%) patients had extraconal and eight (16.3%) had intraconal and orbital apex involvement. Overall, seven of these 25 patients had simultaneous intracranial extension. Intracranial involvement was seen in seven (14.28%) patients. Of these seven patients, four had only dural involvement, one had a small contiguous focus of brain parenchymal involvement adjacent to the cribriform plate, one had a cerebral abscess, and one had cavernous sinus involvement.

Endoscopic sinus surgery (ESS) + sublabial Caldwell Luc approach partial maxillectomy (CWLPM) + endoscopic orbital decompression (EOD) was done in 11 (22.44%) patients, ESS + sublabial Caldwell Luc approach total maxillectomy (CWLTM) + EOD was done in four (8.16%) patients, ESS + CWLPM was done in 13 (26.53%) patients, ESS + endoscopic medial maxillectomy (EMM) + EOD was done in nine (18.36%) patients, ESS + EMM was done in seven (14.28%) patients, ESS alone was done in three (6.14%) patients, and ESS + EOD + craniotomy was done in one (2.04%) patients. No surgery was done in one (2.04%) case, as the patient was not fit for surgery due to poor pulmonary function.

Histopathological examination of the intraoperative biopsy specimen showed broad aseptate fungal hyphae in all 49 cases. Fungal smear showed broad aseptate fungal hyphae in 40 (81.63%) cases, septate fungal hyphae in one (2.04%) case, and no fungal elements in eight (16.33%) cases. Fungal culture grew *Zygomycetes* species in 24 (48.98%) cases, *Aspergillus fumigatus* in five (10.20%) cases, Candida in two (4.08%) cases, and no growth was seen in 18 (36.74%) cases.

Of the 49 patients, 40 received amphotericin-B (AMB), 28 (57.14%) received amphotericin-B lipid emulsion (AMBLE), and 12 (24.48%) were given liposomal amphotericin-B (LAMB) at a dose of 5 mg/kg body weight per day. In total, nine (18.36%) patients did not receive any form of AMB. The total cumulative dose of AMB ranged from 50 mg to 5,050 mg, with a median and mode of 3,000 mg. Retrobulbar injection of AMB was given to two (4.08%) patients with orbital apex involvement. Posaconazole (PCZ) was given in tablet form at a loading dose of 300 mg twice a day, followed by 300 mg once daily to 45 (91.83%) patients. The majority of them, 36 (73.46%), received it as sequential or step-down treatment after AMB therapy, whereas nine (18.36%) received it as standalone therapy. The total cumulative dose of PCZ ranged from 3,300 mg to 27,000 mg, with a mean of 17,495 mg. The duration of treatment ranged from 11 days to 90 days, with a mean of 58 days. Isavuconazole (ISCZ), up to a total cumulative dose of 2,000 mg, was given as salvage therapy to one patient after AMB therapy. Only three patients received AMB as the standalone treatment.

The mean duration of hospital stay was 15 days, with a range of one to 30 days. All patients had regular endoscopic debridement after surgery once a week for one month, followed by once every two weeks till complete clinical and endoscopic resolution of the disease. All patients were followed up for a minimum of one year, except those who died. Immediate (one month, three months) and long-term (six months and one year) follow-up data after treatment are shown in Table 4.

**Table 4:** Follow-up data (N = 49).

Post-treatment follow-up	Cured (A), n (%)	Stable disease (B), n (%)	Unstable disease (C), n (%)	Total cumulative deaths (D), n (%)	Successful outcome (A + B), n (%)	Unsuccessful outcome (C + D), n (%)
1 month	28 (57.14%)	16 (32.65%)	2 (4.08%)	3 (6.12%)	44 (89.79%)	5 (10.20%)
3 months	39 (79.59%)	3 (6.12%)	0 (0%)	7 (14.28%)	42 (85.71%)	7 (14.28%)
6 months	40 (81.63%)	2 (4.08%)	0 (0%)	7 (14.28%)	42 (85.71%)	7 (14.28%)
12 months	41 (83.67%)	1 (2.04%)	0 (0%)	7 (14.28%)	42 (85.71%)	7 (14.28%)

One month after treatment, there were three (6.12%) deaths, two (4.08%) had unstable disease, 16 (32.65%) had residual but stable disease, and 28 (57.14%) were disease-free (cured). All three deaths occurred within the first 15 days after treatment due to septic shock or sudden cardiac arrest.

By the end of three months, there were four more deaths, making it a total of seven (14.28%) deaths, three (6.12%) had residual but stable disease, such as residual osteomyelitic bone of the cribriform plate region or maxilla or body of zygoma, and 39 (79.59%) were disease-free (cured). Among these four deaths, one was due to simultaneous lung involvement with poor lung capacity and lack of fitness for surgery, receiving only antifungal therapy. The second was due to a sudden cardiac arrest at home in a patient with stable but residual disease. The third was due to septic shock and cardiac arrest in the perioperative period after the second surgery (craniotomy) for extensive osteomyelitis of the frontal bone. The fourth was due to an unstable disease with cerebral abscess and a second COVID-19 infection.

By the end of six months, there were no further deaths, two had residual but stable disease in the form of osteomyelitic bone of the cribriform plate in one, body of zygoma in another, and 40 were disease-free (cured). During this time, one patient who had residual osteomyelitic bone of the maxilla was cured from the disease with sequential debridement.

By the end of one year, only one patient had residual disease in the form of osteomyelitic bone of the zygoma with discharging sinus, and 41 were disease-free. This one patient persistently refused any second surgery. The patient who had residual osteomyelitic bone of the cribriform plate also got cured. The all-cause mortality rate was 6.12% at one month, 14.28% at three months, and remained the same even after six months and one year. Short-term (three months) and long-term (one year) treatment success was 85.71%.

When the stage of the disease was compared with overall outcomes over time (Table 5), of the total 49 patients, seven (14.28%) had an unsuccessful outcome (death) by the end of three months, with no further change even at six months and 12 months. Among these seven patients, three (42.85%) were in stage IV, two (28.57%) in stage III, and another two (28.57%) in stage II. Thus, patients with stage III and IV disease together (n = 5) accounted for 71.42% of cases of unsuccessful outcomes. There was no change in successful or unsuccessful outcomes beyond three months.

**Table 5:** Stage of disease versus outcome (N = 49).

Follow-up	Stage of disease	Cured ( A), n	Stable disease (B), n	Unstable disease (C), n	Deaths, n	Cumulative deaths (D), n (%)	Successful outcome (A + B), n (%)	Unsuccessful outcome (C + D), n (%)
1 month	I	1	0	0	0	3 (6.12%)	44 (89.79%)	5 (10.20%)
	II	16	5	0	2			
	III	11	5	1	1			
	IV	0	6	1	0			
	Total	28	16	2	3			
3 months	I	1	0	0	0	7 (14.28%)	42 (85.71%)	7 (14.28%)
	II	20	1	0	0			
	III	15	1	0	1			
	IV	3	1	0	3			
	Total	39	3	0	4			
6 months	I	1	0	0	0	7 (14.28%)	42 (85.71%)	7 (14.28%)
	II	21	0	0	0			
	III	15	1	0	0			
	IV	3	1	0	0			
	Total	40 (81.63%)	2 (4.08%)	0 (0%)	0			
12 months	I	1	0	0	0	7 (14.28%)	42 (85.71%)	7 (14.28%)
	II	21	0	0	0			
	III	15	1	0	0			
	IV	4	0	0	0			
	Total	41 (83.67%)	1 (2.04%)	0 (0%)	0			

Univariate analysis (Table 6) showed no statistically significant association of age, sex, and steroid use with outcomes (successful/unsuccessful) of CAM. Among the comorbidities, only diabetes showed a statistically significant association with the outcome of CAM at one, three, six, and 12 months consistently (p < 0.05). There was a statistically significant association between the number of comorbidities and outcomes of CAM at three months (p = 0.05). There was no significant association between disease laterality and outcomes of CAM. There was a statistically significant association between the extent of disease and outcomes of CAM at three, six, and 12 months (p < 0.05). There was a statistically significant association between the extent of surgery and the outcome of CAM at one and three months (p < 0.05). There was a statistically significant association between the type of antifungal drug regimen used and outcomes of CAM, particularly evident at three, six, and 12 months after treatment (p = 0.001).

**Table 6:** Factors affecting outcome of COVID-19-associated mucormycosis: univariate analysis.

Variable	1 month		3 months		6 months		12 months	
	Chi-square value	P-value	Chi-square value	P-value	Chi-square value	P-value	Chi-square value	P-value
Age	7.3	0.32	8.3	0.41	4.13	0.12	4.13	0.12
Sex	1.25	0.26	0.80	0.64	0.09	0.76	0.09	0.76
Duration of symptoms	7.23	0.38	12.75	0.005	0.18	0.66	0.18	0.66
Disease laterality	2.14	0.14	0.4	0.81	0.21	0.64	0.21	0.64
Steroid use	0.57	0.98	1.36	0.50	0.18	0.66	0.18	0.66
Diabetes	9.13	0.002	7.17	0.007	4.50	0.05	4.50	0.05
Hypertension	0.054	0.81	0.20	0.90	0.12	0.90	0.12	0.90
Coronary artery disease	0.58	0.44	1.32	0.51	0.14	0.70	0.14	0.70
Comorbidity count	2.25	0.68	15.3	0.05	8.58	0.07	8.58	0.07
Extent of disease	3.03	0.69	30.7	0.001	16.3	0.006	16.3	0.006
Extent of surgery	37	0.001	25.3	0.005	6.87	0.33	6.87	0.33
Antifungal drug type	2.92	0.40	36.7	0.001	26.5	0.001	26.5	0.001

A cumulative link mixed-effects model was fitted to assess the association between time, clinical characteristics, and treatment-related factors with ordinal outcomes (death/unstable disease/stable disease/cure) measured longitudinally (baseline, three, six, and 12 months) (Table 7). The model included a random intercept for patient ID to account for repeated measures (n = 49 patients; 196 total observations). The random intercept variance was near zero, indicating minimal subject-level variability after adjusting for fixed effects. There was a statistically significant improvement in outcome over time. Compared to baseline, the odds of being in a higher outcome category (cure) were significantly greater with time. At three months, the odds ratio (OR) was 5.00 and 95% confidence interval (CI) was 1.91-13.17 (p = 0.0015). At six months, OR was 27.47 and 95% CI was 7.56-99.38 (p < 0.001). At 12 months, OR was 27.47 and 95% CI was 7.56-99.38 (p < 0.001).

**Table 7:** Association between time, clinical characteristics, and treatment-related factors with an ordinal outcome (death, unstable disease, stable disease, cure): cumulative link mixed model results. *: Due to model convergence and estimation limitations, levels with only one patient (extent of disease stage I, no surgery categories) were excluded from the cumulative link mixed-effects model. CAD = coronary artery disease; Sx = type of surgery; ESS = endoscopic sinus surgery; EMM = endoscopic medial maxillectomy; EOD = endoscopic orbital decompression; CWLPM = Caldwell-Luc partial maxillectomy; CWLTM = Caldwell-Luc total maxillectomy; CN = craniotomy; AFT = antifungal therapy; PCZ = posaconazole; AMB = amphotericin-B; ISCZ = isavuconazole

Predictor	Estimate	Standard error	Odds ratio	95% confidence interval	P-value	Interpretation
Time (reference = baseline)						
3 months	1.62	0.51	5.0	1.9–13.2	0.0015	↑ odds of a better outcome at 3 months
6 months	3.36	0.65	27.5	7.6–99.4	<0.001	↑↑ odds of a better outcome at 6 months
12 months	3.36	0.65	27.5	7.6–99.4	<0.001	↑↑ odds of a better outcome at 12 months
Demographics						
Age	-0.002	0.02	0.998	0.96–1.04	0.94	No significant effect
Sex (male versus female)	0.33	0.65	1.40	0.39–5.03	0.6	No significant effect
Clinical factors						
Symptom duration	0.006	0.014	1.01	0.98–1.03	0.65	Not significant
Disease side	-0.34	0.42	1.01	0.98–1.03	0.4194	No significant effect
Steroid use (yes)	-0.32	0.55	0.73	0.25–2.11	0.56	No significant effect
Diabetes (yes)	2.51	1.54	16.13	6.04–47.54	0.001	Possible ↑ in the odds of a worse outcome
Hypertension (yes)	-2.39	1.02	0.09	0.01–0.62	0.019	↓↓ odds of a better outcome
CAD (yes)	-0.91	0.99	0.40	0.06–2.25	0.36	No significant effect
Comorbidity count	1.51	0.79	4.53	0.99–20.6	0.057	Possible ↑ in the odds of a worse outcome with more comorbidities
Treatment factors						
*Disease extent: stage II	-0.204	1.58	0.69	0.35–2.09	0.48	No significant effect
Disease extent: stage III	-0.107	1.55	0.75	0.23–2.15	0.59	No significant effect
Disease extent: stage IV	-1.13	1.62	7.13	4.13–21.45	0.001	Possible ↑ in the odds of a worse outcome
Sx: *ESS/ESS + EMM	0.302	1.76	1.35	0.04–43.40	0.86	No significant effect individually
Sx: ESS + EMM + EOD	-0.798	1.75	0.45	0.01–14.01	0.6489	No significant effect
Sx: ESS + CWLPM	0.232	1.735	1.26	0.04–37.85	0.8935	No significant effect
Sx: ESS + CWLPM/CWLTM + EOD	-0.663	1.749	0.52	0.02–15.90	0.7045	No significant effect
Sx: ESS + EOD + CN	-2.041	2.394	0.13	0.00–14.17	0.3939	No significant effect
AFT: AMB alone	3.69	0.73	23.2	6.23–94.7	0.001	↑ odds of a worse outcome
AFT: PCZ alone	-2.12	1.22	0.12	0.01–1.32	0.0826	Trend toward ↓ odds of a worse outcome
AFT: AMB followed by PCZ	-2.26	1.2	0.1	0.01–1.10	0.0597	Trend toward ↓ odds of a worse outcome
AFT: AMB followed by ISCZ	-3.42	2.12	0.71	0.31–1.62	0.1066	No significant effect

There was no statistically significant association between age, sex, duration of symptoms, laterality of disease, steroid use, and CAM outcomes. Among clinical covariates, diabetes was associated with significantly increased odds of adverse clinical progression. Patients with diabetes had approximately 16 times greater odds of worse outcomes compared to non-diabetic individuals (OR = 16.13, 95% CI = 6.04-47.54, p = 0.001). Hypertension was significantly associated with lower odds of improved outcomes (OR = 0.09, 95% CI = 0.01-0.62, p = 0.019). The count of comorbidities showed a statistically significant association with outcome (OR = 4.53, 95% CI = 0.99-20.6, p = 0.057), indicating a possible increase in the odds of worse outcome with more comorbidities.

There was a statistically significant association of only stage IV disease (intracranial extension) with worse outcome of CAM (p = 0.001), while other stages of disease showed no significant effect. No significant association was observed between the extent of surgery and outcomes of CAM.

Antifungal therapy with AMB alone was associated with a statistically significantly worse outcome (OR = 23.2, 95% CI = 6.23-94.7, p = 0.001). PCZ alone showed a trend toward decreased odds of worse outcomes but was not statistically significant (p = 0.08). AMB followed by PCZ therapy showed statistically significant decreased odds of worse outcomes (p = 0.05).

## Discussion

This longitudinal study assessed the clinical outcome predictors for patients with CAM at a 12-month follow-up duration. Our analysis combined univariate chi-square association tests and multivariate longitudinal ordinal logistic regression to holistically address the factors determining clinical progression.

The longitudinal ordinal regression model indicated that relative to baseline, the odds of having a better outcome increased significantly at three months (OR = 5.0, 95% CI = 1.9-13.2, p = 0.0015), with a significant further improvement at six months that was maintained through 12 months (OR = 27.5, 95% CI = 7.6-99.4, p < 0.001). This pattern of progressive improvement indicates that therapeutic interventions in CAM take sufficient time to attain maximal benefit, with the greatest improvements gained between the three and six-month periods.

Neither age nor gender showed strong associations with CAM outcomes in either longitudinal regression or univariate analyses, similar to studies by Pal et al. [6] and Ostovan et al. [7]. This result is different from some prior research [5,8-10] on COVID-19 and mucormycosis individually and indicates that the evolution and responsiveness to treatment in CAM may be fairly homogeneous between demographic subgroups. Nonetheless, our sample size could have limited the ability to recognize subtle demographic impacts, especially age-related gradients of treatment response.

Of the comorbid diseases, diabetes was found to be the strongest and most reliable predictor of adverse outcomes, similar to previous studies [6,11,12]. In longitudinal regression analysis, patients with diabetes showed about 16-fold higher odds of poorer outcomes compared to non-diabetic patients (OR = 16.13, 95% CI = 6.04-47.54, p = 0.001). This was supported by univariate analyses demonstrating significant correlations between diabetes and outcomes at all time points (one month: p = 0.002; three months: p = 0.007; six and 12 months: p = 0.05). The strong and consistent relationship between diabetes and adverse CAM outcomes is most likely due to the multifaceted pathophysiology processes by which hyperglycemia impairs immune function, impairs wound healing, and enhances microvascular dysfunction, which is highly pertinent in the setting of post-COVID-19 immune compromise [1,6,13]. Hypertension was found to have a strong inverse effect on outcomes in this study in the longitudinal model (OR = 0.09, 95% CI = 0.01-0.62, p = 0.019), despite not being evidenced in the individual univariate analyses at any point in time. Hypertension was reported as the second most common risk factor [6] and predictor of CAM outcomes [14] in a few studies similar to our study. Hence, the effect of hypertension interactions over a longer duration was not captured as precisely in a straightforward univariate manner. Notably, our analysis identified a borderline significant relationship between comorbidity burden and outcomes (longitudinal regression: OR = 4.53, 95% CI = 0.99-20.6, p = 0.057; univariate analysis at three months: p = 0.05), implying worse outcomes with a higher number of comorbidities. Steroid use did not show any significant effect on the outcomes of CAM in our study, although 79% of patients used steroids for COVID-19. Our results are similar to the systematic review and meta-analysis of 958 cases of CAM done by Özbek et al. [14]. Several studies [15-19] have shown steroid use during COVID-19 to be a risk factor for CAM. Thus, although steroid use was a risk factor for CAM, it may not have a significant effect on the outcomes of CAM.

Symptom duration had mixed effects on outcomes across analysis methods, with a significant univariate relationship only at the three-month time point (p = 0.005) and no effect in longitudinal models. This indicates that although symptom duration might have an effect on intermediate recovery trajectories, other variables take over its significance in predicting long-term outcomes.

Disease extent had a significant impact on outcomes in both analytical strategies, with stage IV disease (intracranial extension) having especially strong correlations with poorer clinical progression. This emphasizes the prognostic value of baseline disease severity. The value of early diagnosis in CAM, i.e., before widespread tissue invasion, is reinforced by these results. Our results are similar to the studies by Hoenigl et al. [20], Dravid et al. [21], and Manesh et al. [5], where the mortality of CAM was higher with intracranial extension.

Surgical debridement as an adjunct to antifungal therapy was associated with better outcomes in mucormycosis [6,22]. In our study, the effect of the extent of surgery on CAM outcomes exhibited a time-dependent trend, with strong associations only in univariate analysis at one month (p = 0.001) and three months (p = 0.005), but weakening at six and 12 months (p = 0.33). This time-dependent trend indicates that surgical technique likely plays a determining role in early recovery patterns but becomes less determinant of long-term outcomes. Antifungal treatment after surgery and control of comorbidities may ultimately play a more significant role in achieving final clinical resolution.

The selection of the antifungal drug regimen was an important predictor of clinical outcomes. The longitudinal model indicated antifungal therapy with AMB alone was related to much poorer outcomes (OR = 23.2, p = 0.001). PCZ alone or AMB followed by PCZ therapy showed trends toward better outcomes, with the latter regimen showing borderline statistical significance (p = 0.05). These relationships were well-supported by univariate analyses at three, six, and 12 months (all p = 0.001). Our results are similar to the multicentric study by Patel et al. [9], where sequential therapy with AMB followed by triazoles showed better outcomes in CAM. These results should be interpreted with caution, as there were few patients (three) who received AMB alone, and due to the lack of AMB availability during the COVID-19 pandemic, there were interruptions in treatment. Due to a shortage of AMB, patients with early-stage disease might have received PCZ alone, leading to selection bias.

The all-cause short-term (three months) and long-term (one year) mortality rate was 14.28% and the treatment success rate was 85.71%. The mortality rate of CAM in this study was lower than many of the studies, which reported mortality rates ranging from 44% [6] to 26% [12]. A recent systematic review on CAM reported a mortality rate of 34% [6]. Early detection, prompt surgical debridement, and sequential antifungal therapy with AMB followed by PCZ might have helped in achieving successful outcomes and a low mortality rate in our study.

Study limitations

Some study limitations must be noted. Our sample size (n = 49) limited statistical power, especially for identifying smaller effect sizes and interactions between predictors. The ordinal outcome measure, though clinically useful, may fail to capture subtle dimensions of patient symptomatology or functional status. Moreover, treatment allocation was not randomized, with the potential for selection bias in therapeutic comparisons.

## Conclusions

Short-term (three months) and long-term (one year) treatment success of CAM was 85.71% with a mortality rate of 14.28% in our study. Diabetes and hypertension are significant negative predictors of CAM, warranting close monitoring and intervention. Pretreatment baseline disease extent, particularly intracranial extension, adversely affects the outcomes of CAM, highlighting the importance of early diagnosis and treatment. Surgical technique likely plays a determining role in early recovery patterns, but becomes less determinant of long-term outcomes. Drug regimens and comorbidity burden may affect outcomes, but require larger studies for confirmation. Post-treatment improvement in the outcomes of CAM occurs over time, with the greatest improvement between the three and six-month periods, emphasizing the need for continuous and long-term follow-up of these patients for at least six months.
